# Increasing Testing Options for Key Populations in Burundi Through Peer-Assisted HIV Self-Testing: Descriptive Analysis of Routine Programmatic Data

**DOI:** 10.2196/24272

**Published:** 2021-09-30

**Authors:** Tiffany Lillie, Dorica Boyee, Gloriose Kamariza, Alphonse Nkunzimana, Dismas Gashobotse, Navindra Persaud

**Affiliations:** 1 Family Health International 360 Washington, DC United States; 2 Family Health International 360 Dar es Salaam United Republic of Tanzania; 3 Family Health International 360 Bujumbura Burundi

**Keywords:** HIV, HIV self-testing, key populations, case finding, ART initiation, Burundi

## Abstract

**Background:**

In Burundi, given the low testing numbers among key populations (KPs), peer-assisted HIV self-testing (HIVST) was initiated for female sex workers (FSWs), men who have sex with men (MSM), and transgender people to provide another testing option. HIVST was provided by existing peer outreach workers who were trained to provide support before, during, and after the administration of the test. People who screened reactive were referred and actively linked to confirmatory testing, and those confirmed positive were linked to treatment. Standard testing included HIV testing by clinical staff either at mobile clinics in the community or in facilities.

**Objective:**

This study aims to improve access to HIV testing for underserved KPs, improve diagnoses of HIV serostatus among key populations, and link those who were confirmed HIV positive to life-saving treatment for epidemic control.

**Methods:**

A descriptive analysis was conducted using routine programmatic data that were collected during a 9-month implementation period (June 2018 to March 2019) for peer-assisted HIVST among FSWs, MSM, and transgender people in 6 provinces where the US Agency for International Development–and US President’s Emergency Plan for AIDS Relief–funded LINKAGES (Linkage across the Continuum of HIV Services for KP Affected by HIV) Burundi project was being implemented. Chi-square tests were used to compare case-finding rates among individuals who were tested through HIVST versus standard testing. Multivariable logistic regression was performed to assess factors that were independently associated with HIV seropositivity among FSWs and MSM who used HIVST kits.

**Results:**

A total of 2198 HIVST kits were administered (FSWs: 1791/2198, 81.48%; MSM: 363/2198, 16.52%; transgender people: 44/2198, 2%). HIV seropositivity rates from HIVST were significantly higher than those from standard testing for FSWs and MEM and nonsignificantly higher than those from standard testing for transgender people (FSWs: 257/1791, 14.35% vs 890/9609, 9.26%; *P*<.001; MSM: 47/363, 12.95% vs 90/2431, 3.7%; *P*<.001; transgender people: 10/44, 23% vs 6/36, 17%; *P*=.50). Antiretroviral therapy initiation rates were significantly lower among MSM who were confirmed to be HIV positive through HIVST compared to those among MSM who were confirmed to be HIV positive through standard testing (40/47, 85% vs 89/90, 99%; *P*<.001). No significant differences in antiretroviral therapy initiation rates were found between the FSW and transgender groups. Multivariable analyses among FSWs who used HIVST kits showed that being aged ≥25 years (adjusted odds ratio 1.9, 95% CI 1.4-2.6) and having >8 clients per week (adjusted odds ratio 1.3, 95% CI 1.0-1.8) were independently associated with HIV seropositivity.

**Conclusions:**

The results demonstrate the potential effectiveness of HIVST in newly diagnosing underserved KPs and linking them to treatment.

## Introduction

### Background

In October 2016, following the World Health Organization (WHO) recommendations, HIV self-testing (HIVST) was included in the Burundi national testing guidelines for key populations (KPs), including female sex workers (FSWs) and men who have sex with men (MSM), to help achieve the UNAIDS (The Joint United Nations Program on HIV/AIDS) first 90 goal—to have 90% of all people living with HIV know their status [1,2]. In addition, the Burundi HIV 2018-2022 National Strategic Plan puts greater emphasis on KPs than past plans and sets the country’s own *90* goals: 90% of KPs should be reached with prevention messages and access to prevention commodities, 90% should know their HIV status, 90% of those diagnosed with HIV should have access to antiretroviral therapy (ART), and 90% of those on ART should be virally suppressed [3]. Achieving ambitious targets like these requires that HIV programs find new ways to identify individuals who are HIV positive and initiate them on treatment.

The HIV epidemic in Burundi is considered a low-prevalence mixed epidemic. HIV prevalence among the general population in 2017 was estimated at 1%; however, recent studies have found significantly higher HIV prevalence among KPs [4]. Data from the 2014 Priorities for Local AIDS Control Efforts study showed an HIV prevalence of 21.3% among FSWs and 4.8% among MSM [5]. In addition, testing rates in both populations were low; 43% of FSWs and 32% of MSM reported that they had tested for HIV in the past year [5]. In Burundi, sex work and homosexuality are criminalized, which increases stigma and discrimination, harassment, and arrests among KPs [6,7]. Laws and prevailing cultural and social norms that stigmatize KPs not only infringe on their rights but also amplify risk and vulnerability and limit their access to HIV services [6-8]. Therefore, public health programs must find unique and innovative ways to offer differentiated service delivery (DSD), both to address the unique challenges experienced by KPs and to provide individuals with a range of options that meet their needs.

The Burundi Ministry of Health and the National AIDS and Sexually Transmitted Infections Program, supported by the US President’s Emergency Plan for AIDS Relief and the US Agency for International Development through the LINKAGES (Linkages across the Continuum of HIV Services for KPs Affected by HIV) project [9], drafted an implementation plan in 2018 for HIVST in Burundi. HIVST with OraQuick (OraSure Technology, Inc) [10] was initiated in June 2018 for FSWs, MSM, and transgender people to provide another testing option. HIV testing using rapid tests was offered in the community and facilitated by trained medical staff before HIVST. With the introduction of HIVST, lay workers, for the first time, were able to offer peers HIV testing in their preferred locations in the community and at convenient times. The aim was to improve access to HIV testing to underserved KPs, increase HIV positivity rates, and link those who were confirmed HIV positive with life-saving treatment for epidemic control.

A growing body of evidence shows that HIVST is highly acceptable, feasible, convenient, and viewed as more confidential than standard testing services among harder-to-reach and higher-risk populations, including KPs [11-19]. According to early systematic reviews, HIVST, both supervised and unsupervised, had high acceptability among participants [18] and was preferred over standard testing as it was convenient and private [15,17]. A Nigerian study [12], which explored the uptake of HIVST among MSM, found in a survey conducted 3 months after HIVST kits were distributed that almost all the men had used the kit, and most reported that the test was easy to use, confidential, and convenient. Another study among MSM in Uganda found that HIVST was viewed as more confidential and easier to use, and the men appreciated that the results were provided quickly compared with standard testing services [16]. In a study of FSWs in Kenya [13], women found HIVST to be acceptable and accessible. In numerous studies, people have generally preferred HIVST over conventional testing strategies for its convenience and confidentiality, which has increased access to testing services [12,15-17,19].

However, peer-reviewed HIVST literature has raised implementation concerns such as the lack of pre- and posttest counseling [15,17], lack of immediate emotional support and barriers to successful linkage and referral to other needed services [14,16,17,20,21], concern that the test provides unreliable results for those unfamiliar with its administration, and possible user error for those who choose unassisted HIVST [15,16,22,23]. Choko et al [23] found that about 10% of Malawian participants did make some administrative errors in using the kits and that the same percentage required extra help in performing the test. Another study found that first-time testers were less confident about accessing follow-up support compared with previous testers after a reactive test [14].

Numerous studies have concluded that more information, research, and stronger programming to ensure linkage to confirmatory testing and treatment after HIVST is necessary [11-13,17,18,20,21,23-26]; however, few have actually explored this relationship [11-13,20,21,25,26]. Varying rates of linkage to confirmatory testing and treatment were found in studies that used both assisted and unassisted HIVST [11-13,20,25-27]. One systematic review and meta-analysis on unsupervised HIVST among MSM in high-resource countries reported a range of linkage to care from 31.3%-100% [20]. There were a couple of studies that explored the relationship between unsupervised HIVST and linkage to treatment in KPs in Sub-Saharan African countries [11,12]. Both had high linkage rates; however, one had active follow-up and access to a KP-friendly drop-in-center component [12], and the other was not statistically significant from the standard service [13]. There were several studies that described a peer-assisted HIVST model [18,23,25,26]; however, only two have explored the relationship between peer-assisted HIVST and linkage to other services [21,22]. Both studies were from Asia, targeting KPs; one demonstrated a high linkage rate, and the other had no linkage evidence [25,26].

### Objectives

There is a dearth of data describing which HIVST models targeted to KPs in Africa can be effective in closing gaps in testing and treatment. This study aims to improve access to HIV testing for underserved KPs, improve diagnosis of HIV serostatus among key populations, and link those who were confirmed HIV positive with life-saving treatment for epidemic control. We describe our experience with peer-assisted HIVST distribution and implementation among FSWs, MSM, and transgender people in Burundi and compare HIV seropositivity and ART linkage among people screened through HIVST with those screened during standard HIV testing services (HTS).

## Methods

### Geographic Locations

A descriptive analysis was conducted on routine programmatic data that were collected during a 9-month implementation period (June 2018 to March 2019) for peer-assisted HIVST where LINKAGES operated: Bujumbura Mairie (urban), Bujumbura Rural (periurban), Kayanza (periurban and rural), Ngozi (periurban and rural), Kirundo (periurban and rural), and Gitega (urban). The 6 provinces were selected through the US President’s Emergency Plan for AIDS Relief strategic planning processes based on national and subnational epidemiological data and stakeholder consultation. In each province, the selection of communes (a lower administrative unit) for implementing HIVST was based on project-specific mapping and size estimation, routine program monitoring data on testing, and in-country consultation.

### Implementation of Standard HIV Testing by CBOs

The CBOs had previously established outreach programs that delivered services to KPs in a variety of hot spots (geographic areas where KPs were present and where high-risk behaviors were practiced), such as karaoke bars, short-term guest houses, massage parlors, and truck stops. The CBOs hired, trained, and paid peer outreach workers (POWs) to provide KPs with prevention services and referrals to testing for HIV and sexually transmitted infections. The POWs hired by CBOs were recruited from identified hot spots, and selection criteria included identifying as an FSW, a man who has sex with men, or a transgender person; the willingness to work on an HIV project; good communication and leadership skills; and the ability to motivate peers to seek health services. LINKAGES conducted programmatic hot spot mapping and size estimation per venue, and POWs were selected to cover a specific number of venues. POWs had a list of peers that they provided HIV outreach services to, and they usually conducted outreach services on weekends during evening hours when the maximum number of KPs were available. POWs chose their own hours but were supervised and coached by the CBO staff on planning their outreach schedule on a monthly basis.

Clinical staff provided standard testing services (blood-based testing) in the community through mobile testing and in the health facilities. Staff who provided services in the mobile and health facilities followed the national HIV testing algorithm, using a rapid HIV blood-based test. The CBOs also used the programmatic mapping and size-estimation results as well as the peer educators’ information on how many FSWs, MSM, and transgender people needed an HIV test to schedule the mobile testing unit’s monthly operational calendar. POWs also referred KP peers to the facility for HIV testing and other services based on need (ie, HIV testing every 3 months and sexually transmitted infection symptoms) and demand.

### HIVST Implementation Through POWs

The OraQuick HIV self-test kit from OraSure Technologies, with 93% sensitivity and 99% specificity [28,29], was used in the LINKAGES KP program. A total of 87 POWs were trained to conduct peer-assisted HIVST. In the HIVST strategy, trained POWs increased awareness about HIVST among their peers (defined as someone in the same KP group), discussed the benefits of HIVST and reduced the misconceptions about the test, assessed peers’ eligibility for HIVST (ie, not tested in the last 3 months), demonstrated how the HIVST kit was administered, provided abbreviated pre- and posttest counseling, supported the administration of the test, managed the screening results and ethical issues, and provided referrals for follow-up services. The KP peer chose the location and time that they preferred to take the HIVST. Then, the peer self-administered the test with the POW sitting by them to support the individual in taking the sample, timing, and reading the results. The POW would offer posttest counseling and assistance to access other preventative services or confirmation testing based on the results of the test. POWs received a stipend of BIF 30,000 (US $15.30) per month for their involvement in the HIVST program.

The POWs were asked to reach out to other KPs in their communities who would benefit from HTS. The POWs used standardized screening forms to determine peer eligibility for HIVST kits. To be eligible for standard or self-testing, the peer needed to be a KP at high behavioral risk and not tested in the last 3 months. High behavioral risk was determined based on affirmative responses to any of the following questions: having sex with a man in the last month (MSM), receiving money or gifts for sex (MSM and FSW), having >8 sexual partners in the last week (FSW), not using condoms consistently (MSM and FSW), and consuming alcohol and drugs regularly (MSM and FSW). The screening tool for standard testing asked about KP status but not about the number of sexual partners in the last week, consistent condom use, or regular alcohol or drug use. Eligible KP members who were already accessing standard testing services were encouraged to continue testing through this modality every 3 months or to use HIVST if that was preferable. High-risk KP members who were never tested or tested rarely (defined as not tested in the last 6 months) or requested HIVST were offered HIVST. None of the patients who had already accessed standard testing or were rarely or never tested were denied an HIVST kit if they wanted to be tested through this modality. Individuals who did not meet the criteria for participating in HIVST, including those who already knew they were HIV positive, were linked to other HIV services based on need. Once KP peers accepted HIVST, the POWs provided peer-assisted testing.

If the test was nonreactive, peers were offered prevention services, such as risk reduction messages, condoms, and lubricants, and asked to get tested for HIV within 3 months if the risk behaviors continued. All those who had a reactive screening test were either accompanied by the POW or referred to a facility of their choice for confirmatory testing. Individuals with a confirmed positive test result were provided counseling and offered same-day treatment. POWs were supported and mentored by supervisors to ensure that they provided effective counseling and demonstrated good communication skills, could successfully administer the test and interpret the test results, and were able to link their peers to other health services. At the end of the training, HIVST kits were provided to CBOs and POWs to distribute to and assist high-risk and eligible peers.

### Target Populations

The target populations for HIVST implementation were FSWs, MSM, and transgender people. FSWs were defined as women who were aged at least 18 years and who received money or goods in exchange for sexual services, either regularly or occasionally. MSM were defined as men who engaged in sexual relations with other men in the last 12 months. Transgender people were those whose gender identity and expression did not conform to the norms and expectations traditionally associated with the sex assigned to them at birth and included transgender women and men.

### Data Collection and Management

During service delivery, paper-based data collection tools adopted from the LINKAGES global guidance [30] were used by the outreach staff to screen if individuals were members of a KP group and captured data on HIV risk, sociodemographic information, HIVST results, results of follow-up confirmatory tests, and services received by those who were HIV positive. As HIVST was peer assisted, the result of the HIVST was recorded immediately in the main HIVST register. All other services received, including acceptance of a confirmatory test among those who had a reactive screening results and enrollment in treatment, were recorded in the same register. At the end of the 9 months of implementation, data were stripped of all personal identifiers and entered into an electronic database by dedicated program data clerks. The monitoring and evaluation officer reviewed the accuracy of the data, compared the paper records with the information entered in the database, and guided data entry clerks to make corrections where necessary.

For this analysis, the HIVST program database was reviewed, and data were extracted for selected variables, including location, age, KP type, ever tested, tested in the last 6 months, exchange sex for money, number of clients per week (if FSW), condom use, alcohol use, HIVST screening results, confirmatory testing and results, and treatment initiation for all those who received an HIVST kit during the 9-month period. Data on standard testing clients were obtained from the project’s aggregated report for each month during the same period of HIVST implementation. The monthly aggregate reports were made by summarizing client-level data collected using standardized paper-based data collection tools.

### Data Analysis

Descriptive analysis was conducted initially to describe case-finding rates; links to treatment; and sociodemographic, sexual, and health-seeking behaviors of those who screened reactive via HIVST. HIV seropositivity rates and links to treatment were compared among KPs who were screened using HIVST with those tested using standard methods. When comparing HIV seropositivity rates among FSWs and MSM who used HIVST kits with those tested using standard methods, stratified analysis was conducted to adjust the outcome for age. Among FSWs and MSM who used HIVST kits, bivariate analyses were first used to identify sociodemographic characteristics and sexual and health-seeking behaviors associated with HIV seropositivity. Multivariable logistic regression was then used to determine factors independently associated with HIV seropositivity. Variables that were significant in the univariate analysis and geographic area of residence were included in the logistic regression model. The chi-square test or Fisher exact test (when the expected cell value was <5) was used to test for differences in proportions for categorical variables. The two-tailed Student *t* test was used to test for differences in the means of continuous variables. For all comparisons, *P*=.05 was considered significant. All analyses were performed using Stata 14 (StataCorp LLC).

### Ethical Issues

Following local and international norms, all individuals who presented at a testing service and requested an HIV test received either abbreviated pre- and posttest counseling from a POW during HIVST or pre- and posttest counseling from a clinical staff member during standard HIV testing. All KPs provided oral informed consent before the test was conducted. The authors had no access to individual identifying information of those who received either HIVST or standard testing as all data were extracted from a database that contained no personal identifiers. The request to conduct secondary analysis of program data that did not contain any personal identifying information was reviewed by the institutional review board of the Family Health International 360 and was classified as nonhuman-subjects research.

## Results

### HIVST Distribution and User Characteristics

From June 2018 to March 2019, a total of 2198 HIVST kits were distributed and used (FSWs: 1791/2198, 81.48%; MSM: 363/2198, 16.52%; transgender people: 44/2198, 2%). The distribution of the kits was unequal across the 6 geographic areas; of the 2198 kits distributed, 1090 (49.59%) were distributed in Bujumbura Mairie, the capital, and 50 (2.27%) kits in Gitega (Table 1). The mean age of the individuals using the self-test kits was 27 years (SD 7.6). Of the total number of users, 60.05% (1320/2198) were receiving an HIV test for the first time, and 7.05% (155/2198) had been tested for HIV at least once but not in the last 6 months (Table 1)*.*

**Table 1:** Characteristics of those who used self-test kits (N=2198).

Indicator			FSW^a^ (n=1791)		MSM^b^ (n=363)		Transgender people (n=44)		All key populations (n=2198)
**Test kits used, n (%)**			1791 (100)		363 (100)		44 (100)		2198 (100)
	Bujumbura Mairie	819 (45.7)		230 (63.4)		41 (93.2)		1090 (49.6)	
	Bujumbura rural	28 (1.6)		21 (5.8)		3 (6.8)		52 (2.4)	
	Gitega	44 (2.5)		6 (1.7)		0 (0)		50 (2.3)	
	Kayanza	446 (24.9)		49 (13.5)		0 (0)		495 (22.5)	
	Kirundo	43 (2.4)		21 (5.8)		0 (0)		64 (2.9)	
	Ngozi	411 (22.9)		36 (9.9)		0 (0)		447 (20.3)	
Age using self-test kits (years), mean (SD)			27.0 (7.5)		29.60 (8.0)		28.2 (5.5)		27.4 (7.6)
**HIV testing status, n (%)**									
	First-time testers	1013 (56.6)		272 (74.9)		35 (79.5)		1320 (60.1)	
	Previously tested more than 6 months ago	105 (5.9)		46 (12.7)		4 (9.1)		155 (7.1)	
	Previously tested less than 6 months ago	673 (37.6)		45 (12.4)		5 (11.4)		723 (32.9)	

^a^FSW: female sex worker.

^b^MSM: men who have sex with men.

### HIVST Cascade Outcomes

Of the total number of KPs, 16.65% (366/2198) were reactive to HIV screening on HIVST, 95.9% (351/366) sought confirmatory testing, 89.5% (314/351) were confirmed to be HIV positive, and 95.9% (301/314) were initiated on treatment.

HIV seropositivity rates were significantly higher among those tested through HIVST compared with standard testing for FSWs (adjusted odds ratio [aOR] 1.7, 95% CI 1.4-1.9) and MSM (aOR 2.7, 95% CI 1.9-3.9) but not among transgender people. ART initiation rates were lower among MSM who tested through HIVST than those who tested through standard testing (40/47, 85% vs 89/90, 99%; *P*<.001; Table 2).

**Table 2:** HIV seropositivity rates from HIVST^a^ compared with standard testing for FSWs^b^, MSM^c^, and transgender people in Burundi, June 2018 to March 2019 (N=14,274).

Indicator and testing model			FSWs		MSM		Transgender people		All KPs^d^
**Number of KP members confirmed HIV positive**									
	HIVST, n (%)	257 (14)		47 (13)		10 (23)		314 (14)	
	Standard testing, n (%)	890 (9)		90 (4)		6 (17)		986 (8)	
	OR^e^ (95% CI)	1.7 (1.4-1.9)^f^		2.7 (1.9-3.9)^f^		1.5 (0.4-5.5)		1.9 (1.6-2.2)	
	*P* value	<.001		<.001		.50		<.001	
**Number of KP members linked and initiated on ART^g^**									
	Linked from HIVST, n (%)	251 (98)		40 (85)		10 (100)		301 (96)	
	Linked from standard testing, n (%)	864 (97)		89 (99)		6 (100)		959 (97)	
	OR (95% CI)	1.2 (0.5-3.8)		0.1 (0.0-0.5)		1		0.6 (0.3-1.4)	
	*P* value	.61		<.001		—^h^		.21	

^a^HIVST: HIV self-testing.

^b^FSW: female sex worker.

^c^MSM: men who have sex with men.

^d^KP: key population.

^e^OR: odds ratio.

^f^Adjusted for age.

^g^ART: antiretroviral therapy.

^h^Not available (*P* value cannot be calculated because cell has a value of 0).

### User Characteristics Affecting Reactive Results

The multivariable logistic regression model among FSWs indicated that being aged ≥25 years (aOR 1.9, 95% CI 1.4-2.6) and having >8 clients per week (aOR 1.3, 95% CI 1.0-1.8) were significantly associated with a reactive HIVST. Among MSM, being aged ≥25 years and having an HIV test in the last 6 months were significantly associated with HIV seropositivity on bivariate analyses; however, these associations disappeared in the multivariable logistic model. Among MSM, living in Ngozi was significantly associated with a reactive HIVST in the multivariable model (aOR 4.3, 95% CI 1.8-10.5; Table 3).

**Table 3:** Sexual and health-seeking behaviors associated with a reactive HIVST^a^ result (N=2198).

Characteristics and categories			FSW^b^													MSM^c^										
			HIVST outcome confirmed positive, n (%)		Unadjusted					Adjusted					HIVST outcome confirmed positive, n (%)				Unadjusted					Adjusted		
					OR^d^ (95% CI)		*P* value		OR (95% CI)			*P* value						OR (95% CI)			*P* value		OR (95% CI)			*P* value
**Age (years)**			N/A^e^		N/A		<.001		N/A			<.001		N/A				N/A			.03		N/A			.08
	<24	83 (10.2)		1.0 (reference)		N/A		1.0 (reference)			N/A		7 (7)				1.0 (reference)			N/A		1.0 (reference)			N/A	
	>25	174 (17.8)		1.9 (1.4-2.5)		N/A		1.9 (1.4-2.6)			N/A		40 (15.9)				2.5 (1.1-5.7)			N/A		2.2 (0.9-5.1)			N/A	
**Number of clients per week**			N/A		N/A		.005		N/A			.03		N/A				N/A			N/A		N/A			N/A
	<8	139 (12.5)		1.0 (reference)		N/A		1.0 (reference)			N/A		N/A				N/A			N/A		N/A			N/A	
	>8	117 (17.4)		1.5 (1.1-1.9)		N/A		1.3 (1.0-1.8)			N/A		N/A				N/A			N/A		N/A			N/A	
**Ever tested for HIV**			N/A		N/A		.46		N/A			N/A		N/A				N/A			.37		N/A			N/A
	No	151 (15)		1.0 (reference)		N/A		N/A			N/A		33 (12.5)				1.0 (reference)			N/A		N/A			N/A	
	Yes	105 (13.7)		0.9 (0.7-1.2)		N/A		N/A			N/A		14 (16.3)				1.4 (0.7-2.7)			N/A		N/A			N/A	
**Recently tested in the last 6 months**			N/A		N/A		.54		N/A			N/A		N/A				N/A			.02		N/A			.10
	No	165 (14.6)		1.0 (reference)		N/A		N/A			N/A		36 (11.8)				1.0 (reference)			N/A		1.0 (reference)			N/A	
	Yes	91 (13.7)		0.9 (0.7-1.2)		N/A		N/A			N/A		11 (24.4)				2.4 (1.1-5.2)			N/A		2.3 (0.9 6.4)			N/A	
**Do you use condoms for every sexual intercourse?**			N/A		N/A		N/A		N/A			N/A		N/A				N/A			N/A		N/A			N/A
	Never	49 (14.8)		1.0 (reference)		N/A		N/A			N/A		22 (19.8)				1.0 (reference)			N/A		N/A			N/A	
	Sometimes	199 (14.3)		0.9 (0.7-1.4)		.83		N/A			N/A		21 (9.4)				0.4 (0.2-0.8)			.009		N/A			N/A	
	Always	5 (9.4)		0.6 (0.2-1.6)		.30		N/A			N/A		3 (21.4)				1.1 (0.3-4.3)			.89		N/A			N/A	
**Geographical areas**																										
	Bujumbura Mairie	139 (17)		1.0 (reference)		N/A		1.0 (reference)			N/A		19 (8.7)				1.0 (reference)			N/A		1.0 (reference)			N/A	
	Bujumbura	9 (32.1)		2.3 (1.0-5.2)		.04		1.9 (0.8-4.4)			.12		7 (33.3)				5.2 (1.9-14.6)			.001		2.6 (0.8-8.9)			.13	
	Gitega	10 (23.8)		1.5 (0.7-3.2)		.26		1.2 (0.6-2.6)			.64		1 (16.7)				2.1 (0.2-18.9)			.51		1.4 (0.1-13.1)			.79	
	Kayanza	48 (10.8)		0.6 (0.4-0.8)		.003		0.6 (0.4-0.8)			.002		7 (14.3)				1.7 (0.7-4.4)			.24		1.9 (0.7-4.8)			.19	
	Kirundo	5 (11.9)		0.7 (0.3-1.7)		.39		0.7 (0.3-1.8)			.45		3 (14.3)				1.7 (0.5-6.5)			.40		1.6 (0.4-6.1)			.48	
	Ngozi	46 (11.2)		0.6 (0.4-0.9)		.008		0.6 (0.4-0.9)			.01		10 (27.8)				4.0 (1.7-9.6)			.002		4.3 (1.8-10.5)			.001	

^a^HIVST: HIV self-testing.

^b^FSW: female sex worker.

^c^MSM: men who have sex with men.

^d^OR: odds ratio.

^e^N/A: not applicable.

## Discussion

### Principal Findings

The aim of the HIVST strategy was to increase access to HTS among KPs, increase new HIV diagnoses, and ensure effective treatment initiation once a reactive test was confirmed positive. Through this strategy, we were able to provide access to HTS to many MSM, FSWs, and transgender people not previously tested and who would likely not test given the stigma and discrimination from health care workers and lack of convenience and privacy at facilities. It was found that individuals initially screened through HIVST had a higher HIV positivity rate compared with those who chose standard HIV testing. ART initiation was similar between standard testing and HIVST for both FSWs and transgender people. However, lower rates of ART initiation were observed among MSM newly diagnosed through HIVST compared with standard testing. HIVST is an additional testing option that can help reach the most marginalized. It is an approach to support the achievement of the first and second 95 UNAIDS goals.

Offering peer-assisted HIVST to KPs provided the opportunity for those who never tested or rarely tested to access HTS and learn their serostatus. For the first time in Burundi, POWs were able to offer and aid peers in taking an HIV test in a private and confidential location of their choice and at a day and time convenient to them. Several studies supported Burundi’s peer-assisted network implementation strategy by showing that KP members prefer to receive HIVST services at the community level through existing KP-friendly drop-in centers, peers, or hot spots [16,19,31]. In our study, of those who accepted HIVST, over half of the FSWs (1013/1791, 56.56%) had never tested previously, and 74.9% (272/363) of the MSM and 80% (35/44) of transgender people had never accessed HTS, and approximately 12.7% (46/363) of MSM and 9% (4/44) of transgender people who had tested previously had not tested in the last 6 months, which is consistent with other study findings [19,20,27]. Although new users did not have a statistically significant higher HIV positivity rate compared with those who tested in the last 6 months, the high number of new users who accessed HTS through HIVST is a public health success, given the low testing rates among KPs in Burundi [5]. The introduction of HIVST in Burundi was successful in its ability to expand HIV testing options to higher-risk populations who were not otherwise served by current HIV services and could learn their serostatus, which could overcome the underdiagnosis of HIV among KPs.

The HIVST strategy enabled the program to reach underserved populations and achieved higher HIV positivity rates compared with standard testing for FSWs and MSM. This could be attributed to several factors. First, KPs had more options for community-based testing, which they preferred over clinic-based models. This is especially important in settings such as Burundi, where high levels of stigma and discrimination exist. It is well documented that Burundi’s KP community has high HIV prevalence levels, low testing rates, and high levels of criminalization and discrimination, which directly affect service use [6,8]. Second, HIVST (oral fluid based) is less invasive than standard testing (blood based). Third, HIVST is peer-assisted through POWs, whereas standard facility-based testing requires interaction with non-peer clinical staff. Fourth, with HIVST, KPs can choose when and where to take the HIVST without the need to visit a health facility. Not all KP individuals are comfortable accessing standard medical services even if they are provided by KP-friendly staff. Finally, POWs used a more targeted screening tool to determine eligibility for HIVST compared with standard testing; however, no one was denied an HIVST if it was their preferred testing method. The high proportion of KPs who were never or rarely tested and chose HIVST is a demonstration that this testing modality is meeting an unmet need for a certain segment of KPs. Other studies also found that those who accessed HIVST had a higher HIV positivity rate compared with standard testing services [19,24,32]. A meta-analysis of two randomized controlled trials found that offering HIVST to MSM doubled the likelihood of an HIV-positive diagnosis [24]. Many HIV programs struggle to engage and test KPs despite high HIV prevalence, given the multiple barriers to services such as stigma, discrimination, and criminalization—indicating service delivery gaps [33]. The Burundi HIVST achievement demonstrates the need for programs to constantly assess their current service delivery packages and creatively expand them based on specific population needs.

The sexual and health behaviors of those who had reactive results through HIVST were explored to better understand the risk characteristics underlying higher positivity rates. Among FSWs, having >8 clients per week and being aged >24 years were factors affecting higher HIV positivity rates, which is consistent with the overall HIV epidemic in Burundi [4]. Among MSM, there were no health-seeking or behavioral factors that were statistically significant between those who were nonreactive and those who were reactive; however, a large number of MSM used condoms inconsistently, which is a high-risk behavior associated with greater infection rates. MSM living in Ngozi also had higher HIV positivity rates, which may warrant expanding and strengthening HIV services in the province.

A key concern in the literature is the ability for those who test reactive through HIVST and are subsequently confirmed HIV positive to be effectively linked to care and treatment services [17,20,21]. A systematic review and meta-analysis of HIVST among MSM reported that linkage to care ranged from 31.3%-100%, indicating programmatic gaps and reluctance to seek additional services [20]. When reviewing other peer-assisted HIVST models [18,23,25,26], it was noted that only a couple of studies explored the relationship with linkage to confirmation testing and treatment services and had varying levels of success [25,26]. Studies investigating unsupervised HIVST with linkage to confirmation testing and treatment services also showed varying achievement levels [12,13,20,21]. In this study of peer-assisted HIVST, the rate of linkage to treatment was lower overall among KPs who tested positive via HIVST compared with standard testing; however, when data were disaggregated by population, there was no difference in FSWs and transgender people, but linkage to ART was lower among MSM. LINKAGES started implementing the HIV program for KPs in August 2016 and, historically, had a high treatment initiation rate, >90%, for FSWs, MSM, and transgender people [34]. Therefore, when ART initiation rates among MSM who were newly diagnosed through HIVST was 85%, it was a concern, given the consistently high linkage rates over the previous 2 years. Since the inception of the program, a key component to LINKAGES was training and hiring strong KP community members to become peer navigators (PNs) [35]. PNs provided ART adherence support in the community, acted as advocates for HIV-positive KPs within facilities, and strengthened the client-patient relationship. PNs were also present during standard community-based testing provided by clinical staff. Then, when someone tested HIV positive through the community-based testing site, peer navigation support was offered, and, if accepted, the PN then provided accompaniment to the clinic for ART initiation. With the advent of HIVST, PNs were trained on HIVST and asked to provide it within their peer networks. Then, if a peer was reactive, PNs offered the same linkage and adherence support as they had historically provided and recorded the linkage and support through the monitoring system. Therefore, although there could have been a measurement issue within the community-based HIVST program in its inability to accurately track an individual from a reactive test to confirmation testing and ART initiation, the program did not give this concern as much weight, given PNs’ historical role in monitoring. Then, as confirmatory testing for both HIVST and standard testing were mostly conducted within the same facilities, no measurement gap could be attributed at this stage. This study offers additional information and recommendations on how expanding testing modalities within an HIV program can meet an existing unmet need in populations.

Currently, in Burundi, HIV treatment is only available at registered facilities, including MSM drop-in centers. LINKAGES sensitized the providers at supported clinical sites on the provision of KP-friendly services using the Health4All training curriculum [36]. The training focused on how to provide quality, stigma-free HIV services, including clinical competencies for each of the KP groups. In addition, LINKAGES worked with the local and district health, law enforcement, and administrative authorities on stigma and discrimination reduction to create a more favorable environment for KP health programs. The activities to strengthen KP-friendly services within the LINKAGES program were successful, with 97.1% (864/890) MSM who were newly diagnosed with HIV through standard services initiating treatment. The rate of MSM who were newly diagnosed and initiated on treatment from standard services was substantially higher than the rate of MSM newly diagnosed and initiated on ART from HIVST (40/47, 85% of newly diagnosed linked to ART). For achieving the three 90 UNAIDS goals, service delivery will have to become more diversified and client-centered. Otherwise, HIV transmission and acquisition will persist in certain segments of the population, and epidemic control will remain a challenge. The WHO recommends that treatment programs offer DSD and emphasizes the need to have various options so that individuals can choose which model best meets their needs. Therefore, although the standard testing and facility-based ART model in Burundi is well suited for most MSM, there remains a service delivery gap for a certain segment of MSM clients.

The Burundi example confirms that HIVST is a highly acceptable and feasible testing modality among KPs. Key concerns about HIVST in the literature include the lack of pre- and posttest counseling and an individual’s ability to successfully self-administer the test. The Burundi strategy addressed these concerns by providing peer-assisted HIVST kits with trained POWs. The trained POWs provided abbreviated pre- and posttest counseling, aided peers in test administration, and supported those who tested reactive to confirmatory testing or facilitated confirmatory testing in the community. The implementation strategy allowed the program to successfully track individuals through the HIVST monitoring cascade from use, test result, confirmation testing, and ART initiation if the individual was confirmed HIV positive. The program did not offer unsupervised HIVST during this implementation period, given the challenge in offering follow-up services, such as counseling and ART initiation, to those who may have a reactive result. However, after more than a year of implementation, the program is planning to offer unsupervised testing to sexual partners of KP members who are HIV positive and reluctant to access standard testing.

### Limitations

The implementation and analysis were not without limitations. First, there were differences in the two populations who chose either HIVST or standard testing, such as HIVST users having a high rate of never tested or not tested in the last 6 months. The authors acknowledge these differences but note that the 2 groups chose the testing modality based on personal preferences. Those who chose HIVST also chose not to access existing standard services, which is a lesson learned for present and future HIV programs targeting KP. In the future, the authors can conduct additional analyses on the similarities and differences between the 2 users to better understand users’ key characteristics that may affect the testing modality of choice. Second, the authors were not able to determine whether the high rates of testing among never testers were because of increased access or HIVST alone. Third, as the analysis was based on programmatic data among people reached by POWs in specific geographic areas, the findings may not be generalizable to all FSWs, MSM, and transgender people in Burundi. Fourth, for HIVST, client records were kept in an electronic database; however, for standard testing, all client records were maintained in a paper-based system. As a result, we were unable to comprehensively compare those tested with HIVST versus standard testing across variables such as date of last HIV test, HIV risk, and health care–seeking behaviors. In addition, the high positivity rates among KP members who used HIVST kits could possibly be affected by repeat testers who already knew their HIV-positive status. Therefore, when asked if they knew their HIV serostatus by the POW, they could have falsely reported their testing history and serostatus as they wanted to confirm their HIV-positive serostatus. We attempted to reduce this possibility by comparing newly diagnosed individuals among HIVST and standard testing with existing programmatic records and could not find duplicate records; however, this possibility cannot be totally eliminated. KP members who tested nonreactive could also have used more than one HIVST kit over the recruitment period, although it should be noted that most KP members reported never or rarely tested (not tested in the last 6 months). Finally, higher positivity rates could be an effect of the POW networks and their ability to access higher-risk and more highly infectious individuals; however, to further explore this relationship, additional analysis will have to be performed. Future analyses could explore the relationship between higher positivity rates and individual POWs to see if there is a correlation. If there is a correlation, then programs should attempt to saturate the social and sexual networks of those POWs to further increase HIV diagnoses.

The authors wished to explore the effectiveness of how adding a new HIV testing modality to the Burundi KP program could reach individuals who were not currently accessing services because of structural, social, site, or personal barriers. Given the WHO call for DSD, the authors wished to demonstrate how adding one new HIV testing modality could significantly improve service uptake among those who chose not to access existing services. The authors acknowledge the limitations of programmatic data, which restrict their ability to explore all differences in the 2 groups; however, given the success of HIVST among KPs in Burundi in both case finding and treatment uptake, they felt it important to share with other public health professionals that HIVST is a means to achieve the first and second 90 in a low-resourced setting among a highly stigmatized and criminalized population.

### Conclusions

The results demonstrated the effectiveness of the peer-assisted HIVST implementation model in identifying newly diagnosed HIV positive cases in Burundi. More widespread implementation of HIVST within other high-risk populations and other geographical areas could accelerate progress toward epidemic control.

## Abbreviations

aOR: adjusted odds ratio
ART: antiretroviral therapy
CBO: community-based organization
DSD: differentiated service delivery
FSW: female sex worker
HIVST: HIV self-testing
HTS: HIV testing service
KP: key population
LINKAGES: Linkages across the Continuum of HIV Services for Key Populations Affected by HIV
MSM: men who have sex with men
PN: peer navigator
POW: peer outreach worker
UNAIDS: The Joint United Nations Program on HIV/AIDS
WHO: World Health Organization
